# Supplementary material for: Are carbon emissions trading and green financial instruments synergistic? -Comprehensive quantitative research based on content analysis
Source: PLoS One. 2024 Mar 7;19(3):e0298601. doi: 10.1371/journal.pone.0298601 (PMC10919601; doi:10.1371/journal.pone.0298601)
Supplement: S1 Data — (ZIP) [file pone.0298601.s001.zip › Data/地级市Z_F工作报告.zip!/╜¡╦╒/╨∞╓▌╩╨/└┤╫╘╣1⁄2╓┌║┼í╛┬φ┐╦╩2╛▌═°í┐_2011.docx]

各位代表：

现在，我代表市人民政府向大会作工作报告，请予审议，并请市政协各位委员和其他列席人员提出意见。

一、“十一五”及2010年经济社会发展回顾

2006年以来，在省委、省政府和市委的正确领导下，全市上下坚持以邓小平理论和“三个代表”重要思想为指导，深入贯彻落实科学发展观，围绕振兴老工业基地，突出抓好经济建设、中心城市建设、和谐社会建设和政府自身建设，圆满完成了“十一五”规划及2010年目标任务。

——五年来，全市经济发展跃上新台阶

综合经济实力大幅提升。全市主要经济指标增速连续五年高于全省平均水平。2010年全市地区生产总值（预计，下同）达2800亿元，人均地区生产总值超过3万元，分别比上年增长14%和14.7%左右，均比2005年翻一番；财政一般预算收入实现222亿元，比上年增长35.2%，比2005年翻两番。县域主要经济指标增速连续四年高于全市平均水平，铜山、邳州、沛县跨入“全国百强县”行列，“突破睢宁”和“丰县崛起”战略扎实推进，新沂工商税收总量进入苏北县级前十强。民营经济快速发展，实现增加值占地区生产总值比重达65%。全市小康社会建设达到序时进度，四大类18项25个指标中有20个实现目标值。

经济结构调整取得重大进展。集中力量推进“三重一大”，2010年全市固定资产投资完成2049.3亿元，比上年增长26.1%，其中工业投入首超千亿；五年累计完成固定资产投资6638亿元，是“十五”时期的3.3倍。中能多晶硅、卡特彼勒大型挖掘机、徐工斗山发动机和大吨位起重机、华润电力等一批重大项目相继建成或开工建设。产业层次明显提升，我市荣获“中国工程机械之都”称号，建成产量亚洲第一、世界第三的多晶硅生产基地。全市新增首发上市企业3家，徐工集团实现整体上市。2010年全市装备制造、食品及农副产品加工、商贸物流旅游三大主导产业均超过千亿元规模，煤盐化工、冶金、建材三大传统产业完成产值1299.7亿元，新兴产业和高新技术产业产值分别比上年增长85.4%和90%。服务业加快发展，城市中心商圈、新城区商务中心、高铁生态商务区建设强力推进，4家省级和33家市级现代服务业集聚区建设进展顺利。社会消费品零售总额比上年增长19%，是2005年的2.4倍。

开放型经济开创崭新局面。2010年全市实际到帐注册外资和出口总额分别达10.13亿美元、26.3亿美元，比上年增长43.3%和73.6%；五年累计实际到帐注册外资、出口总额分别达30亿美元、93亿美元，是“十五”时期的2.5倍和4.5倍，对外经济合作领域进一步扩大。强力推进徐州经济技术开发区“二次创业”，争先进位步伐持续加快，综合实力、园区面貌不断实现新跨越，7家省级开发区、6家南北共建园区建设快速推进，沛县新型铝材产业园等6个园区成为省级特色园区。

——五年来，城乡面貌发生历史性变化

中心城市建设全面拉开。强化规划引领，修编完善各类城市规划220项，完成114个镇的镇村布局和村庄建设规划。强化功能性项目建设，2010年实施的149项城建重点工程有84项顺利竣工，五年累计投入750多亿元完成270多项城建重点工程，老城区面貌显著提升，新城区建设初具规模，高铁站区开发扎实推进。强化综合交通枢纽地位，五年累计完成交通投资200亿元，徐济高速徐州段建成通车，实现所有县（市）区半小时上高速目标，京沪高铁徐州段基本建成。强化城市空间拓展，主城区面积扩大到3037平方公里，县（市）城镇基础设施建设不断加强，全市城市化率达53%。强化城市管理，构建了条块联动的“大城管”体制，“数字化城管”系统试运行，淮海路成功创建省级示范路，改造新建14个农贸市场，展览馆夜市、开明市场搬迁改造顺利完成，城区环卫保洁、绿化养护市场化运作实现全覆盖。

“三农”工作力度持续加大。各级财政五年累计投入“三农”资金259亿元，带动社会资金投入176亿元。粮食总产七年连增，2010年达89.5亿斤，设施农业和高效农业面积占比保持全省第一，农业产业化综合水平走在全省前列，铜山区获批为国家现代农业示范区。“农村新五件实事”和新一轮“农村六大实事工程”扎实推进，建成260个新农村示范村和600个环境综合整治示范村，新建改建村综合服务中心1092个；全市水利建设投资累计达71亿元，是“十五”时期的2.5倍；解决了271.5万农村人口饮水安全问题；新建农村公路3948公里；219个经济薄弱村债务全部化解；91.8万农村贫困人口实现脱贫。

生态环境建设有力推进。全市环保投资五年累计达136亿元。截至去年底，城市生活污水集中处理率、生活垃圾无害化处理率均达85%以上，城区空气质量优良天数占全年比例连续五年稳定在85%以上，城市集中式饮用水源地连续6年100%达标；在全省率先关闭了立窑水泥生产线及小造纸、小焦化企业，对市区140多家重度污染企业进行了搬迁改造，北区环境整治力度进一步加大，全面完成省下达的“十一五”节能减排和淮河流域水污染防治任务。全市造林面积96万亩、森林覆盖率达29.5%，城市新增绿地6300公顷、绿化覆盖率达40.6%；实施了6万亩采煤塌陷地复垦治理，九里湖湿地公园基本建成，中德共建徐州生态示范区工作进展顺利。

——五年来，和谐社会建设取得突破性进展

民生民计显著改善。城乡居民收入保持较快增长，2010年城市居民人均可支配收入和农民人均纯收入分别达20840元和7780元，比上年增长11%和12%，是2005年的1.9倍和1.8倍。就业创业扎实推进，五年累计新增城镇就业57万人，失业人员再就业28.2万人，期末城镇登记失业率为2.6%，“零就业家庭”持续动态清零；建成国家级大学生创业基地，全市大学生创业企业超过500家。社会保障体系逐步健全，五年累计投入社会保障资金369亿元、年均增长26%，其中各级财政投入131亿元，职工和居民医保医疗费报销比例分别达80%和60%以上，新农合参合率达99%以上，新农保参保覆盖率和基础养老金发放率均达98%以上，城乡低保实现应保尽保、保障标准居苏北首位；社会救助水平稳步提高，慈善事业蓬勃兴起，困难群众、残疾人等特殊群体的基本生活保障逐步强化。住房保障力度持续加大，全市建成369万平方米的经济适用房和廉租房；市区完成408万平方米棚户区拆迁，回购和建设389万平方米居民安置房，超额完成省下达任务；对177个老居民小区进行了综合整治，受益群众达60万人。努力稳定消费品价格，及时落实物价补贴制度，有力保障了困难群众的基本生活。

各项社会事业健康发展。2010年，全市专利申请量和授权量分别达9000件和4620件，五年累计完成量是“十五”时期的5.1倍和4.1倍；五年累计完成科技成果728项，争取国家和省科技计划项目1107项，高层次人才队伍建设成效明显。全市义务教育覆盖率达100%，实施了义务教育学校绩效工资制度，初中毕业生升学率、高中阶段优质学校比例分别比2005年提高17和30个百分点，高校在校生由11万人跃升至17万人，四个主城区通过省教育现代化验收。市直文化体制改革改制基本完成，演艺集团和文化产业集团成功组建，公共文化事业和文化产业发展进一步加快。基本公共卫生服务项目实现全覆盖，全市60%的地区实施了基本药物制度，药品价格降低50%左右。全民健身活动蓬勃开展，竞技体育实力稳步提升，8人15次获得世界冠军。人口与计划生育综合改革深入推进，出生人口性别比失衡状况得到初步遏制。第2次经济普查圆满完成，第6次人口普查顺利推进。双拥工作水平进一步提升，荣膺全国双拥模范城“六连冠”。援川任务在全省率先完成，援疆工作有序推进。高质量完成参与世博会的各项任务。人防、民兵、民族宗教、档案、外事、侨务、台湾事务、统计、广播电视、新闻出版、住房公积金管理、无线电管理、淮塔管理、防震减灾、气象、妇女儿童、青少年、关心下一代、老龄等各项事业都取得了长足发展。

社会保持持续和谐稳定。深入开展法治徐州、平安徐州建设，建立完善领导干部接访下访和包挂重点信访案件等制度，着力加强基层基础和政权建设，和谐社区建设稳步推进，全面实施重大事项社会稳定风险评估，逐步完善社会治安大防控体系和社会矛盾大调解机制，我市连续三年被评为全省社会治安综合治理先进市。不断强化安全生产专项整治，各类事故起数和死亡人数分别比2005年下降37.3%和24.2%，安全生产环境明显改善。

——五年来，政府自身建设得到全面加强

行政效率效能持续提升。市级政府机构改革顺利完成，县（市）区政府和乡镇机构改革稳步实施。第一批生产经营类事业单位改革基本结束，社会公益类和行政执行类事业单位改革积极推进。市直国有企业改革改制基本完成。行政审批事项削减39%，市行政服务中心许可项目入驻率、窗口现场办结率分别达94%和91%。“万人评议机关”等活动深入开展，政务公开逐步深化，市、县两级行政权力实现网上公开透明运行。

推动发展的体制机制更加健全。针对国际金融危机带来的严重影响，建立健全了服务企业制度和中小企业金融顾问制度。在严格保护耕地的前提下，强化集约节约用地，为发展提供了有效的用地保障。建立“松绑放权、自我提拔”的激励机制，开发区的自主发展活力明显增强。落实积极的财政政策，减税让费扶持企业。实施“引银入徐”战略，成功引进5家股份制商业银行和8家农村商业银行分支机构。

依法行政和勤政廉政建设不断加强。严格落实行政执法责任制，建立健全120余项内控制度，顺利完成“五五”普法规划。自觉接受市人大及其常委会的法律监督和工作监督、市政协及社会各界的民主监督，政府决策的科学化、民主化、法制化水平进一步提高。深入推进“廉洁徐州”、“勤廉徐州”创建，广泛开展系列主题教育活动，政府工作人员的依法行政意识和拒腐防变能力明显增强。

各位代表！刚刚过去的五年，是我市加快推进科学发展、跨越发展、和谐发展的五年，是人民收入水平显著提高、生活环境显著改善、生活质量显著提升的五年。特别在多年来奠定的坚实基础上，去年全市经济社会发展实现了一系列令人振奋的重大突破，市区部分行政区划调整顺利完成，徐州经济开发区升格为国家级经济技术开发区，观音机场晋升为国家级对外开放航空口岸，亿吨大港和湖西航道改扩建工程开工建设，国家环保模范城市创建通过复核验收。这些重大事项的完成，为我市的长远发展开辟了广阔的空间。

回顾“十一五”的奋斗历程，我们深切地体会到，全市上下坚定不移地抓住用好各种重大战略机遇，坚定不移地推进“三重一大”建设，坚定不移地提升城市综合竞争力，坚定不移地持续改善民生，坚定不移地推动争先进位、竞相发展，不仅取得了经济社会发展的丰硕成果，而且创造了许多推动科学发展的宝贵经验。五年来的巨大成就，深深凝聚了全市960万人民的心血和汗水，充分体现了各位人大代表、政协委员和社会各界人士的监督、支持与帮助。在此，我代表市人民政府，向全市各族人民群众、向人民解放军驻徐部队、武警官兵和公安民警，向各民主党派、工商联、人民团体和无党派人士，向支持徐州现代化建设的香港特别行政区同胞、澳门特别行政区同胞、台湾同胞、海外侨胞和各界人士，向投身徐州经济社会发展的国内外投资者和企业家，表示衷心的感谢！

在肯定成绩的同时，我们也清醒地看到，我市经济社会发展中依然存在着不少困难和问题，主要是：经济结构性矛盾还没有得到根本解决，高新技术产业和服务业占比还低于全省平均水平；开放型经济整体发展水平不高，出口对经济发展的拉动力较弱；城市化进程相对较慢，与小康社会目标要求存在着较大差距；政府职能尚需进一步转变，行政效能仍要进一步提升；农民收入持续较快增长的难度增加，部分群众生活还比较困难，城市交通拥堵、物价上涨压力加大、住房价格过快增长等，对改善民生民计带来了新的挑战。对此，我们将采取有力措施加以解决。

二、“十二五”时期的奋斗目标和战略部署

今后五年，是我市加快振兴老工业基地、全面建设小康社会的关键时期，是深化改革开放、加快转变经济发展方式的攻坚阶段。综观国内外形势，我国经济发展长期向好的趋势没有改变，国家将实施积极的财政政策和稳健的货币政策，省委、省政府振兴徐州老工业基地战略深入推进，沿海开发和长三角发展步伐不断加快，我市率先进入高铁时代，加上近年来相继建成一大批重大项目，这些都为我市发展带来新的机遇。同时我们也深刻认识到，当前世界经济发展的不确定因素依然较多，经济环境更趋复杂；受国内流动性旺盛、国际输入型通胀等因素影响，国内通胀预期增加，人民币升值压力加大，信贷资金供应趋紧，资源环境约束进一步强化，对我市经济发展特别是经济结构调整提出了更高的要求。根据“十二五”期间的形势和任务，按照市委十届十次全会通过的“十二五”规划建议，市政府组织制定了“十二五”规划纲要（草案），提出了今后五年的总体要求、主要目标和重大战略措施。

今后五年全市经济社会发展的总体要求是：高举中国特色社会主义伟大旗帜，坚持以邓小平理论和“三个代表”重要思想为指导，深入贯彻落实科学发展观，以推动跨越发展、建设美好徐州为主题，以加快转变经济发展方式为主线，以深化改革开放为动力，着力实施经济结构战略性调整，着力推动城乡区域协调发展，着力加强环境保护，着力保障和改善民生，统筹推进经济、政治、文化、社会和生态建设，巩固扩大应对国际金融危机冲击的成果，全面振兴徐州老工业基地，全面建成小康社会，全面建设特大型区域性中心城市，开创科学发展、跨越发展的新局面。

今后五年全市经济社会发展的主要目标是：（1）经济综合实力迈入全国发达地区行列。全市地区生产总值确保超过5000亿元，人均地区生产总值超过5万元，财政一般预算收入超过500亿元。（2）转变经济发展方式取得重大进展。高新技术产业产值占工业总产值比重提高到30%以上，服务业增加值占地区生产总值比重力争每年提高1个百分点，研发投入占地区生产总值比重提高到2%以上。（3）改革开放向纵深推进。城乡一体化、行政管理体制、社会管理体制、财税金融体制等重点领域和关键环节改革取得明显进展，全方位对外开放格局基本形成，经济国际化水平明显提高。（4）特大型区域性中心城市建设取得重大突破。城市化水平提高到57%以上，基本建成区域性产业、交通、商贸、教育医疗、物流、旅游、金融、文化“八大中心”。（5）生态文明建设走在全国同类城市前列。全市森林覆盖率达到32%，城市绿化覆盖率达到45%，单位地区生产总值能耗和主要污染物排放强度大幅下降，建成国家森林城市和生态园林城市。（6）建成全面小康社会。2011年以市为单位基本建成全面小康社会，2014年以县为单位建成全面小康社会；城乡居民收入力争六年倍增，2011年确保人均年收入2500元以下的贫困人口全部脱贫。

今后五年，我们将深入贯彻党的十七届五中全会和省委十一届九次全会精神，按照市委十届十次全会的战略部署，把转变经济发展方式和改善民生贯穿经济社会发展的全过程，大力实施科教和人才强市战略，加快促进科教资源转化为经济社会发展的优势；大力实施创新驱动战略，加快建设充满活力的创新型城市；大力实施绿色增长战略，加快构建资源节约型和环境友好型社会；大力实施开放带动战略，积极呼应沿海开发，加快融入长三角；大力实施统筹发展战略，加快推动城乡一体化发展、经济社会协调发展；大力实施民生优先战略，采取更加扎实有力的措施改善民生民计，让改革发展成果更多地惠及于民。

各位代表！立足新的起点，展望美好愿景，我们坚信，只要全市上下共同努力，抢抓新机遇、推动新跨越，一定能够谱写徐州又好又快发展的新篇章！

三、2011年政府工作的主要任务

2011年是实施“十二五”规划的开局之年，也是全面完成老工业基地振兴“三年目标”和以市为单位基本建成全面小康社会的关键一年。做好今年的工作，对于顺利实现“十二五”规划目标，具有极为重要的意义。我们将按照“十二五”规划的总体部署，以扎实有效的措施，全力以赴推动全市经济社会发展再上新台阶。

今年全市经济社会发展的主要预期目标是：（1）地区生产总值增长13%。（2）财政一般预算收入增长18%。（3）全社会固定资产投资（新口径）增长22%。（4）实际到帐注册外资增长15%。（5）自营出口总额增长15%。（6）社会消费品零售总额增长18%。（7）城镇居民人均可支配收入增长12%以上，农民人均纯收入增长12%以上。（8）城镇登记失业率控制在4%以内。（9）高新技术产业产值占规模以上工业的比重达20%，研发投入占地区生产总值的比重达1.6%。（10）节能减排完成省下达任务。（11）人口自然增长率控制在6‰以内。

为实现上述目标，我们将着力抓好六个方面的工作：

（一）以壮大主导产业和培育世界级企业为重点，强力推进产业转型升级

加大对主导产业和世界级企业的培育力度。强化工业主导产业的链条延伸和层次提升，突出抓好工程机械整机制造的大吨位、自动化和关键零部件的配套完善，继续强化传统能源产业转型转移和新能源产业发展壮大，重点推进乳品、饮料、果蔬等食品及农副产品精深加工，力争年内装备制造、能源、食品及农副产品加工业发展规模分别达到1800亿元、1000亿元和1400亿元。突出“上大压小、整合集聚、延伸发展”，加快煤盐化工、建材、冶金三大传统优势产业改造升级，力争年内产业总规模达到1500亿元。加速培育大企业大集团，采取有力措施支持徐工集团、中能硅业加快向世界级企业的目标迈进，努力形成在国内工业经济和全球同行业中的竞争优势；与此同时，引导扶持现有超百亿企业不断增强综合竞争力，力争年内新增2-3家百亿元企业。

加快农业提档升级步伐。全面落实强农惠农各项政策，进一步增加“三农”投入，着力提高农业科技和产业化水平。积极扶持粮食生产，稳定粮食播种面积，支持优质专用粮食产业发展。加快发展现代农业，年内新增高效农业面积100万亩，其中新增设施农业面积20万亩，重点建设20个设施蔬菜、瓜果生产基地和20个规模养殖场，突出抓好国家级和省市级现代农业产业园区建设。大力推进农业产业化经营，加快发展农民专业合作组织，培育形成一批省级农业龙头企业。高度重视农产品市场流通、质量安全和品牌体系建设，不断增强徐州农产品的综合竞争力。积极推进农业机械化，抓好秸秆禁烧和综合利用。全面加强农田水利建设和农业综合开发，完成年度水利投资20亿元，农业综合开发面积达到25万亩。

推动高新技术和新兴产业跨越发展。大力强化政策扶持、重点产业培育和创新载体建设，努力实现高新技术产业和新兴产业产值均增长80%的目标。不断加大科技创新投入，引导金融信贷和社会资金更多地支持创新型经济发展。统筹推进新能源、新材料、新医药、节能环保、物联网、软件与服务外包六大新兴产业发展，把矿山安全物联网产业作为重要突破口，强力推动核心技术向产业化成果的快速转化。突出抓好力晶光电产业园、徐州软件园、安全生产科技产业园等产业载体建设，高标准推进国家级大学生创业园、中国矿大国家级大学科技园、徐师大文化创意产业园等创新型基地建设，全力支持铜山高新技术产业开发区建设，加快实施徐工研究院等一批企业研发机构和自主创新平台建设。

着力打造区域性现代服务业高地。大力推进软件开发、国际服务外包、现代物流、文化动漫、旅游服务和商务中介六大重点现代服务业发展，重点培育20个龙头骨干型和一批销售收入超10亿元的现代服务业企业，高标准抓好4个省级和33个市级现代服务业集聚区建设，推动制造业企业将服务业环节分离或外包。围绕建设淮海经济区第一商圈的目标，加快中央国际广场、苏宁商务广场、金鹰国际二期等重大服务业项目建设，不断提升综合开发和建设水平。围绕建设区域性现代物流中心的目标，加大物流资源重组力度，重点推进八里国际家居交易博览中心、金驹现代物流园、新城区生活物流园等现代物流基地建设。围绕建设区域性旅游中心城市的目标，加快建设彭城欢乐世界、微山湖千岛湿地等重点旅游项目，积极整合全市旅游资源，强化旅游品牌营销和城市形象宣传，推动旅游业大开发、大发展。

大力提升县域经济和城区经济发展水平。突出重大项目建设、主导产业培育、园区经济发展三大重点，推进县域经济和城区经济协调发展。在各县（市）科学选择2-3个主导产业进行重点培育扶持，引导镇域经济错位发展，打造“一村一品”的特色格局，力争“突破睢宁”和“丰县崛起”战略取得更大成效，新沂跻身全国百强县，沛县、邳州在百强县中的位次稳步提升。支持鼓楼区、云龙区、泉山区有重点、差别化地发展现代服务业，推动铜山区、贾汪区产业升级，加快铜山区和老城区一体化进程，促进贾汪区尽快融入主城区发展的大框架。

全力破解瓶颈制约难题。抓住资金、土地、环评等关键环节，扎实做好对内衔接和向上争取各项工作。进一步巩固金融生态环境创建成果，引导金融机构不断扩大信贷规模，健全银政企对接合作平台，大力推动企业上市，年内新增3家首发上市企业，积极拓宽企业债券、短期融资券以及中小企业集合票据等直接融资渠道，有效缓解资金压力。牢固树立保障科学发展、保护耕地红线的“双保”理念，全面落实征地补偿各项政策，维护好被征地农民的合法权益，加大土地违法责任追究和执法奖惩工作力度，积极规范地开展城乡建设用地增减挂钩和“万顷良田建设”等工作。继续强化节能减排对产业优化升级的倒逼机制，坚持源头控制、科技支撑、严格监管，加快主城区工业企业向开发园区集中，大力淘汰落后产能，加强节能新产品、新技术、新工艺的开发应用，严格控制工业“三废”排放，确保完成省下达的节能减排任务。

（二）以狠抓“三重一大”和扩大消费为重点，强力推进三大需求较快增长

坚定不移推进“三重一大”。计划实施重大项目180项，年度投资597亿元；滚动实施超亿元工业项目300项，其中当年竣工120项。扎实推进亿吨大港一期、湖西航道一期、丰沛铁路、宿新高速、海伦哲专用车辆、荧茂触摸屏及保护镜片等45个续建项目，力争早日竣工运营；加快实施徐兰客运专线、南水北调东线一期、中小河流治理、徐工重卡、晶旺光电LED芯片、徐烟技改等95个新上项目，力争加快开工建设；积极抓好城市轨道交通、观音机场二期扩建、徐宿淮盐通铁路、大郭庄机场搬迁、煤基清洁能源等前期推进项目，力争实现突破性进展。

多措并举扩大消费需求。进一步挖掘城乡消费潜力，大力开拓农村市场，继续开展家电、建材、农机具下乡等工作，着力加强“新网工程”、“万村千乡工程”等农村流通网络和服务体系建设，新建100个为农服务中心。积极促进消费结构升级，巩固提升传统消费，稳定住房、汽车等大宗消费，扩大文化消费、老龄消费、旅游消费和健身消费等多样化服务消费。深入开展放心消费活动，加快推进“放心肉”、“放心菜”、“放心粮油”工程，严厉打击扰乱市场秩序的行为，建立健全规范有序的市场体系。

大力提升对外贸易的规模和水平。强化对外贸企业的挂钩服务，帮助企业化解人民币升值等方面带来的出口压力，积极争取更多的企业进入省跨境贸易人民币结算行列。进一步优化出口主体、商品和贸易结构，鼓励民营企业和中小企业扩大出口，抓好工程机械、光伏光电、大蒜、板材等重点出口基地建设。有针对性地引导冶金、煤矿等具有比较优势的企业“走出去”，开展工程承包，建设境外资源基地。

（三）以提升城市功能和城市化水平为重点，强力推进特大型区域性中心城市建设

强化城建重点工程建设。在道路畅通、景观绿化和商贸文化等六个方面，计划实施186项城建重点工程，年度投资262亿元。在老城区，大力推进“市区道路三年畅通”工程，突出抓好三环东路高架快速路、新淮海西路、韩山隧道等道路建设；加快历史文化片区改造，实施回龙窝、快哉亭改造等建设工程；提高城市景观绿化水平，实施云龙湖景观改造、云龙山敞园、科技广场、西苑体育公园等一批景观绿化工程。在新城区，实施中心医院新城区分院、大龙湖五星级酒店、水街等公共服务项目建设。在高铁站区，进一步强化基础设施建设，满足如期通车的全面要求；加快推进道路、东西广场、长途客运站、公交枢纽站、商办服务设施等功能性配套项目建设，打造交通枢纽、生态、商务、居住“四位一体”的城市副中心。统筹抓好铜山区、贾汪区城建重点工程，推进徐贾快速通道、微山湖沿湖大道一期、潘安湖湿地公园、吕梁山风景区等工程建设。

强化县城镇和新农村建设。搞好县、镇、村三级规划衔接，支持五个县城镇加快发展，加强30个重点中心镇建设，协调推进经济发展、环境建设、民生改善等工作，重点加大产业发展和基础设施建设的帮扶力度，加快建设一批工业型、农业型、旅游型、商贸型和综合型中心镇。有序推进农民转市民，推动符合条件的农业转移人口转为城镇居民，力争全市城市化水平较快提升。加强农村基础设施建设，建设农村公路260公里，疏浚县乡河道1200公里，新建50个新农村示范村和100个环境综合整治示范村，解决好50万农村人口饮水安全问题。

强化环境综合治理。推行以“街长制”、“片长制”为载体的“网格化”城市管理，加大对主次干道、窗口地区、背街小巷、居民小区等重点区域的综合整治，确保市区主干道两侧门头店招首次设置规范率达到98%以上，生活垃圾无害化处理率达到90%以上，改造新建一批农贸市场。优先发展公共交通，着力解决行车难、停车难、车辆非法营运等问题。切实加强环境保护和生态文明建设，加大污水管网建设力度，建设并启用丁万河、新城区等7个污水处理厂，大力实施“二次进军荒山”计划和城市增绿工程，确保全市绿化造林8万亩、新增城市绿地1100公顷以上。

强化淮海经济区核心区一体化建设。加快推动淮海经济区列入国家区域发展规划，积极构建沿东陇海线工业走廊，深化完善核心区8个城市的区域合作体系，重点推进交通、产业、市场、旅游等领域的交流合作，扩大区内各城市之间医保联网结算，建立鲜活农副产品流通“绿色通道”，努力在联动发展上迈出更大步伐。

（四）以扩大招商引资和发展园区经济为重点，强力推进对内对外开放和体制机制改革

用更大力度抓好招商引资。围绕主导产业、新兴产业、现代农业和重点现代服务业，强化专业化招商和产业链招商，以徐台直接通航为契机，加大对台招商力度，继续抓好香港、欧盟、长三角、珠三角等境内外招商，力争引进一批投资超10亿元和1亿美元的大项目。精心组织香港及深圳招商周、第14届投资洽谈会暨2011汉文化国际旅游节等两项综合招商活动，以及日韩招商、欧盟招商、第4届现代农业投资推介会、苏台光伏光电产业合作论坛等系列专题招商活动，组织开展重大招商活动绩效评估，加大考核推进力度，提高招商引资活动的实效。

不断增强开发园区的集聚带动能力。加快推动开发区转型升级，引导各开发区重点培育2-3个主导产业，力争在全省开发区排名中有明显进位。大力支持徐州经济技术开发区创建国家级生态园区，办好中德东方鲁尔产业园、高铁生态商务区等专业园区，推进工程机械、光伏光电、现代物流等特色产业园建设。进一步强化各省级开发区和工业集中区的基础设施建设，推动工业项目向开发区和工业集中区集聚；深化南北合作园区共建，在更大范围、更高层次上承接产业转移。把工业开发园区的发展理念引入农业和服务业领域，加大现代农业产业园区和现代服务业集聚区建设力度，全面推进项目集中建设、产业集群发展。

积极稳妥推进各项改革。进一步深化农村改革，探索和建立土地承包管理的长效机制，扩大土地承包经营权登记试点。加大行政审批制度改革力度，全面完成县级政府和乡镇机构改革任务，推进政府部门所办企业改制。继续推进事业单位分类改革，实施绩效工资改革，全面推行岗位设置管理制度。大力实施“引银入徐”战略，加快农信社改制组行步伐，多渠道搭建担保公司、财务公司等非银行金融性机构，规范投融资平台建设和发展。深化市区财政体制改革，强化综合治税。完成市县文化行政管理机构改革、经营性文化事业单位及文艺院团转企改制。深入推进医药卫生体制改革，实现基本药物制度全覆盖。

加快发展民营经济。认真落实国家和省、市鼓励民间投资的政策意见，切实降低民营经济准入门槛，引导民营企业积极融入大企业、大集团，吸引民资进入重大基础设施、重大产业和重大城建及实事项目建设领域。选择100家成长型企业作为重点扶持对象，着力培育壮大铜山机械和食品、邳州板材、丰县电动车、沛县煤化工、新沂农用化工、睢宁纺织、贾汪建材等以民营经济为主体的产业集群，推动民营经济快速发展。

（五）以强化“两增两控”和社会保障为重点，强力推进和谐社会建设

精心抓好“两增两控”。在增加居民收入上，启动实施城乡居民收入六年倍增计划，高度重视农民收入增长问题，大力推动农业、就业、创业、物业“四业富民”，完成8万农村贫困人口脱贫任务；抓好职工工资增加和困难群体帮扶等重点，建立职工工资正常增长机制，提高最低工资标准。在增加就业上，加强劳动技能培训，重点推进困难和特殊群体就业，充分发挥大学生创业的示范带动作用，深入推动全民创业，确保新增城镇就业7.5万人、失业人员就业再就业3.5万人、农村劳动力转移7万人，城镇登记失业率控制在4%以内。在市场物价调控上，加强主要农产品、基本生活必需品、重点生产资料的生产和供应，突出抓好产销衔接和区域合作，扩大由田间到市场的直销规模，努力压降流通成本；加强市场价格监测预警和监督检查，严格控制政府调价项目，建立健全农副产品应急储备制度，确保各项补贴制度落实到位。在住房价格调控上，严格落实各项调控政策措施，完善商品房价格预售和备案制度，加大保障性住房建设力度，年内全市建设4150套经济适用房、586套廉租房、2850套公共租赁房，实施市区200万平方米的城中村及危旧房拆迁改造，对人均住房10平方米以下的家庭实现廉租房保障应保尽保。

着力完善社会保障体系。强化社会保险征缴扩面工作，在扩大覆盖范围、提高统筹层次、实现制度统一等方面迈出新步伐。确保城镇职工养老金按时足额发放、城乡低保应保尽保，城镇居民医保参保缴费率达95%以上，新农合参保率稳定在95%以上，新农保和基础养老金发放基本实现全覆盖，农村五保老人集中供养率达67%以上。高度重视并逐步解决被征地农民的基本生活保障问题。进一步完善对困难群体基本生活的社会救助机制。

统筹抓好各项社会事业。加快教育强市步伐，加大教育投入力度，积极发展学前教育，推动义务教育均衡发展，扩大高中教育优质资源，支持职业教育特色发展、高等教育内涵发展，重视老年教育发展。着力抓好中小学校舍安全工程，逐步解决高中阶段教师收入偏低和偏远农村教师住房等问题。实行高层次人才队伍建设、高科技创新成果转化、高新技术产业发展“三高联动”，分层次、有重点地抓好现有人才的教育培训，大力培养引进紧缺急需人才与创新创业团队。优化医疗卫生资源配置，抓好公共卫生服务项目建设，创建7个省级、20个市级示范镇卫生院，完成标准化村卫生室建设任务。广泛开展群众性精神文明创建活动，大力加强未成年人思想道德建设，进一步提高市民综合素质，确保通过江苏省文明城市复核验收，争创全国文明城市工作先进市。组织实施精品文化培育、公共文化服务、文化遗产保护等工程，加快文化产业集聚区建设，推动文化大市向文化强市转变。积极抓好体育事业，完善公共体育设施，加快奥林匹克体育中心建设，精心做好第18届省运会筹备工作。继续稳定低生育水平，提高出生人口素质，综合治理出生人口性别比偏高问题。扎实推进残疾人创业就业、残疾人学生教育专项补贴等五项工程，加快建设残疾人社会保障体系和公共服务体系。推动双拥工作特色化，确保实现全国双拥模范城“七连冠”。统筹抓好人防、民兵、民族宗教、档案、外事、侨务、台湾事务、统计、审计、广播电视、新闻出版、住房公积金管理、无线电管理、淮塔管理、防震减灾、气象、妇女儿童、青少年、关心下一代、老龄、慈善、援疆等方面的工作。

巩固和谐稳定的社会局面。继续推行领导干部接访下访和重大信访案件包挂制度，健全矛盾纠纷源头预防机制和社会稳定风险评估机制，强化居委会、村委会的基层基础作用，完善社区和农村治安防控体系，健全大排查、大调解、大防控体系，深入推进法治徐州、平安徐州建设，争创全国社会治安综合治理优秀市。强化食品药品安全监管，严格落实安全生产“一岗双责”，抓好专项整治，完善应急管理体系，有效防范各类事故和突发事件的发生。

（六）以创先争优和提高服务水平为重点，强力推进服务型政府和法治政府建设

大力开展创先争优活动。围绕全面振兴徐州老工业基地、基本建成小康社会、加快进入全国发达地区行列，把全市的工作放在全省和淮海经济区的大局中去比较、去衡量，抓住综合竞争力提升和可持续发展的关键性指标，确保全市主要经济指标增速高于苏北、高于周边地区、高于全省平均水平，各县（市）区超过全省县（市）区平均水平，努力做到速度指标确保争先、总量指标奋力进位、结构指标有力提升。在市政府工作部门深入开展对标找差，积极推动各项工作上台阶、上水平。

着力增强服务发展、服务群众的能力。加强学习型、创新型政府建设，增强经济调节和市场监管能力，提高社会管理和公共服务水平，为各类市场主体创造良好的发展环境。完善企业分类调度服务制度，强化督查督办，用创新的举措破解制约发展的各种瓶颈。扎实推进为民兴办的9个方面56件实事项目，加大财政保障和工作推进力度，确保完成年度目标任务。深入推行行政问责制度和绩效管理制度，建立健全行政效能评估机制和投诉处理机制，引导各级干部真正把心思用在干事创业上，切实增强政府及各部门的创造力凝聚力战斗力。

不断提升依法行政水平。深入落实《国务院关于加强法治政府建设的意见》，健全领导干部法律知识学习培训长效机制，完善重大行政决策机制，深化行政权力公开透明运行；建立行政执法自由裁量权基准制度，细化量化行政裁量权，有效避免执法的随意性。认真接受市人大及其常委会的法律监督和工作监督、市政协的民主监督和司法机关依法实施的监督，高度重视新闻舆论监督和社会公众监督，密切与各民主党派的联系，积极支持统一战线工作。

深入推进政府廉政建设。坚持以廉政准则为准绳，严格规范各级领导干部的从政行为；认真落实《关于实行党风廉政建设责任制的规定》，着力解决影响廉政责任制落实的突出问题，切实做到责任明确、考核到位、追究有力。加强惩治和预防腐败体系建设，加大教育、监督、改革、制度创新力度，不断拓展从源头上防治腐败的工作领域；深入开展纠风工作，坚决纠正损害群众利益的不正之风，建设为民、务实、廉洁、高效的公务员队伍，营造风清气正的发展环境。

各位代表！

承继“十一五”发展，再创“十二五”辉煌，是时代赋予我们的光荣使命。让我们紧密团结在以胡锦涛同志为总书记的党中央周围，认真贯彻落实省委、省政府和市委的决策部署，抢抓机遇，乘势而上，为全面完成“十二五”规划各项任务，共同开创徐州更加美好的明天而努力奋斗，以在苏北率先建成全面小康社会的优异成绩，迎接中国共产党成立90周年！
